# Effect of Preablation Glycemic Control on Outcomes of Atrial Fibrillation Patients With Diabetes Mellitus Following Valvular Surgery Combined With the Cox-Maze IV Procedure

**DOI:** 10.3389/fcvm.2022.898642

**Published:** 2022-05-11

**Authors:** Zhan Peng, Rui Zhao, Yuhua Liu, Yunxiao Yang, Xiubin Yang, Kun Hua

**Affiliations:** ^1^Department of Cardiovascular Surgery, Beijing Anzhen Hospital, Capital Medical University, Beijing, China; ^2^National Center for Cardiovascular Diseases, Fuwai Hospital, Chinese Academy of Medical Sciences and Peking Union Medical College, Beijing, China

**Keywords:** glycated hemoglobin, valvular atrial fibrillation, diabetes mellitus, atrial fibrillation recurrence, outcomes

## Abstract

**Background:**

This study was performed to assess the effect of preablation glycemic control on atrial fibrillation recurrence rates after heart valve surgery concomitant with Cox-Maze IV ablation.

**Methods:**

Twelve-month preablation trends in glycemic control were studied. Recurrence and clinical outcome data were obtained during a mean follow-up period of 36.7 ± 23.3 months postablation.

**Results:**

Higher glycated hemoglobin (HbA1c) at the time of ablation was associated with higher postablation recurrence rates. The cumulative atrial fibrillation recurrence-free survival of patients with HbA1c ≥7.5% at the time of operation at 12, 24, 36 and 48 months was 97.1, 78.3, 54.2, and 36.3%, respectively (*P* < 0.001), and 100, 84.9, 37.2, and 16.2% for patients who preoperatively had an upward trend in HbA1c, respectively (*P* < 0.001).

**Conclusion:**

Maintaining a downward trend in HbA1c during the 12-month period before the operation and an HbA1c value < 7.5% at the time of the operation reduced the recurrence of AF among patients who underwent heart valve surgery concomitant with the Cox-Maze IV procedure.

## Introduction

Diabetes mellitus (DM) is significantly associated with an increased risk for developing atrial fibrillation (AF) (1), which is ~40% compared to individuals without DM (2). Furthermore, DM patients with AF have a greater risk for thromboembolic complications, heart failure, all-cause mortality and cardiovascular mortality (3–5). A series of small prospective clinical trials have demonstrated that stricter glycemic control among diabetic patients can reduce the incidence of perioperative AF and improve survival among patients with DM undergoing coronary artery bypass grafting (CABG) (6, 7) or during catheter ablation (8). Surgical Cox-Maze IV ablation is a well-established and effective strategy for valvular disease patients with AF (9). Whether glycemic control alters the recurrence rate of valvular atrial fibrillation (VAF) or outcomes of valvular disease among patients with DM remains unknown, and the association between glycated hemoglobin (HbA1c) targets and atrial fibrillation has not been well investigated. We aimed to further investigate the effect of preablation glycemic control on clinical outcomes of VAF patients with diabetes mellitus following the Cox-Maze IV procedure.

## Methods

### Study Design

This study protocol was approved by the Ethics Committee of Beijing Anzhen Hospital (No. 2020101X) and was in accord with the Declaration of Helsinki. From June 2016 to January 2021, 378 consecutive valvular atrial fibrillation patients with diabetes mellitus underwent the Cox-Maze IV procedure in our center. Among them, 59 patients (15.6%) who underwent concomitant CABG, had fewer than two HbA1c results, were < 12 months preablation, missed one of the three initial follow-up visits or were followed for < 12 months were excluded. Therefore, 319 patients who underwent valvular surgery combined with the Cox-Maze IV procedure were included in the present study.

Perioperative clinical and baseline data were collected from the institutional database system, and follow-up data were obtained using standardized forms during telephone or clinic visits. In-hospital outcomes included surgical mortality (death within 30 days or the rehospitalization for the same condition after operation) and in-hospital morbidity (respiratory complications, infection, re-exploration for bleeding, stroke, renal dysfunction, or myocardial infarction). Respiratory complications included prolonged ventilator support for more than 48 h or pneumonia after surgery. Renal dysfunction was defined as a serum creatinine level increase of more than 50% or the need for continuous renal replacement therapy. Follow-up outcomes included all-cause mortality, recurrence of AF, wound infection, rehospitalization, stroke and hemorrhage. The follow-up period in our study began on the date of the Cox-Maze procedure. All patients were followed for at least 12 months and had scheduled clinic visits at 3, 6, and 12 months at which time a 24 h Holter-ECG recording was performed, and all these results were reviewed by an experienced investigator. The postoperative recurrence of AF was considered to be any documented AF lasting more than half a minute after a 3-month blanking period, which may be called the therapy stabilization period (10), and we prescribed heart rate-controlling drugs if arrhythmia recurred during this period.

### Surgical Techniques and Medical Treatment

All procedures were performed under cardiopulmonary bypass (CPB) with median sternotomy. The energy source used for each procedure was selected at the surgeon's discretion (AtriCure Inc., West Chester, OH, USA and Medtronic). The Cox-Maze IV procedure and valvular operative approach were performed as previously reported (11). Briefly, the ablation of left and right pulmonary vein (PVs) were guided by urine catheter after the cut of the Marshall ligament, and the left atrial (LA) appendage incision to the left superior PV was performed, then the PVs circle ablation and the linear ablation of posterior mitral valve annulus were created after the continuous suture with the LA appendage. Then, the ablation lesion set for the right side were created between the superior and inferior caval cannulation and from the atrial incision to the tricuspid annulus; Additional lines were performed from the caudal end of the incision to the coronary sinus and the inferoseptal commissure. To ensure the transmurality of the Cox-Maze IV procedure, it was repeated 3–4 times for every lesion. Concomitant valve repair or replacement was performed after ablation. Postoperatively, the patients were under 24-h rhythm monitoring and continuous intravenous amiodarone administration. Then, oral amiodarone was administered 200 mg twice a day for 6 months when the patients left the intensive care unit (ICU). The anticoagulation strategy was warfarin combined with low molecular weight heparin in the early postoperative period and warfarin alone after discharge.

### Statistical Analysis

Continuous variables are represented as the mean and standard deviation, and normally distributed variables were compared by using Student's *t*-test. The Mann–Whitney test was used to compare nonnormally distributed data, which are represented as the median and the interquartile range, and the Wilcoxon rank sum test was performed as appropriate. Categorical variables are represented as frequencies and proportions. The chi-square test was performed to analyze categorical variables, and Fisher's exact test was used for smaller samples.

The distribution of glycated hemoglobin (HbA1c) and the trends of preablation glycemic control were analyzed. Participants were divided into five groups according to perioperative HbA1c levels (<4, 4–5.9, 6–7.9, 8–9.9, ≥10%). Cox proportional hazards regression models were used to estimate the hazard ratios (HRs) and their corresponding 95% CIs of the recurrence of atrial fibrillation. The associations between the levels of HbA1c and the recurrence of AF were analyzed by using restricted cubic spline curves on a continuous scale, which were based on Cox proportional models. The analyses were performed with adjustment for age, sex, body mass index, hypertension, recent smoking, history of congestive heart failure, blood urea nitrogen, creatinine, C-reactive protein, and left ventricular ejection fraction. Multivariable adjusted analyses with 4 knots (4, 6, 8, 10%) were used. The test result for nonlinearity was checked first. If the test for nonlinearity was not significant, the test result for overall association and linearity was checked, with a significant result indicating the linear association.

Analysis of variance (ANOVA) was used for normally distributed continuous variables in the multiple group analysis. In addition, Bonferroni corrections were applied for pairwise comparisons. The Kaplan–Meier method and the log-rank test were used to compare the cumulative event rates. To identify the independent predictors of AF recurrence, we developed univariate and multivariate Cox proportional hazards models and reported the hazard ratios (HRs) and 95% confidence intervals (CIs), with adjustment for potential confounding baseline covariates (age, sex, body mass index, hypertension, recent smoking, history of congestive heart failure, blood urea nitrogen, creatinine, C-reactive protein, left ventricular ejection fraction), which may have a prognostic influence on the outcomes of interest. Variables with *P* < 0.05 were considered statistical predictors of AF recurrence. All relevant preablation characteristics were analyzed in the univariate models, and the significant variables were incorporated into the multivariate models. A two-sided *P* < 0.05 was considered statistically significant, and data were analyzed using SPSS 22.0 and Stata/SE 15.1.

## Result

### Baseline Characteristics of the Overall Population

**Figure 1:**
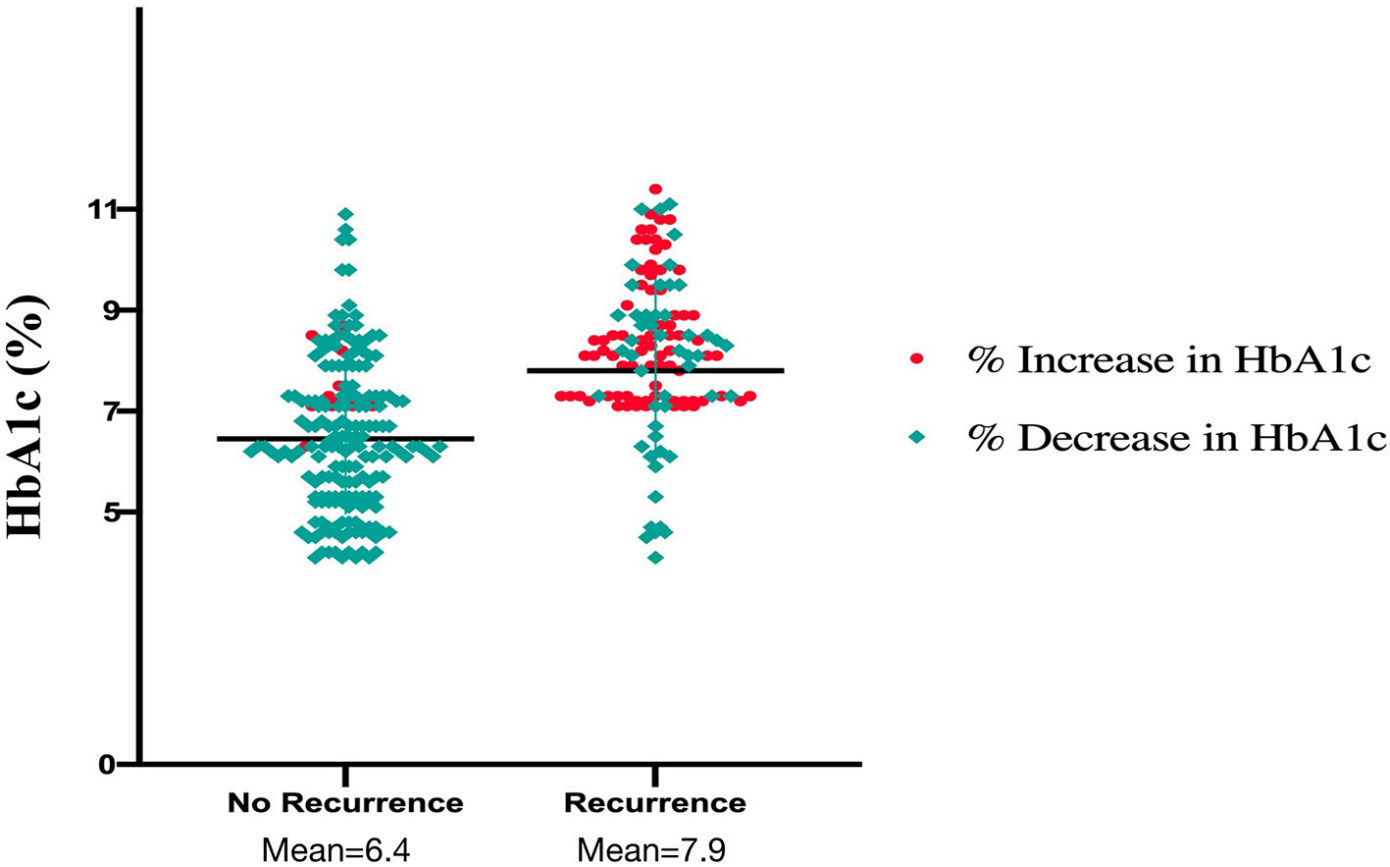
Plasma levels of glycated hemoglobin and the trend of glycated hemoglobin in recurrence and non-recurrence groups.

The mean age of our study cohort was 62.4 years (SD: 7.1). Our study cohort included 160 males (50.2%); moreover, 48 (15.1%) of the patients had type 1 diabetes, and 271 (84.9%) had type 2 diabetes. A total of 111 patients (34.8%) used insulin. Regarding the use of oral hypoglycemic drugs, 163 patients took metformin (51.3%), 65 patients took sulfonylureas (20.4%), 44 patients took dipeptidyl peptidase-4 inhibitors (13.8%), 15 patients took glucagon-like peptide-1 receptor antagonists (4.7%), 11 patients took sodium-glucose cotransporter-2 inhibitors (3.4%), and 13 patients took thiazolidinediones (4.3%). The average perioperative baseline HbA1c was 7.5% for the overall population, 6.4% for patients who had no recurrence of AF, and 7.9% for patients who had AF recurrence. In addition, 233 (73.0%) patients developed a downward trend in preablation HbA1c, and 86 (26.9%) developed an upward trend (Figure 1).

### Dose–Response Analysis of HbA1c Levels With AF Recurrence

**Figure 2:**
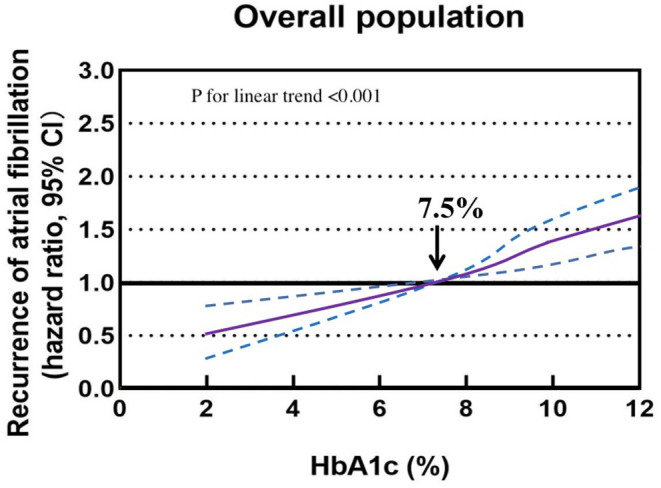
Association of glycated hemoglobin with the recurrence of atrial fibrillation in a restricted cubic spline model. Solid purple lines are multivariable adjusted hazard ratios, with dashed blue lines showing 95% confidence intervals with glycated hemoglobin 6–7.9% as the reference value.

The association of HbA1c levels with the recurrence of AF is shown in Table 1. Compared with participants with HbA1c 6–7.9%, the multivariable adjusted HRs (95% CIs) were 0.58 (0.51–0.72), 0.85 (0.63–0.97), 1.25 (1.03–1.48), and 1.51 (1.23–1.76) for the recurrence of AF in participants with HbA1c <4, 4–5.9, 8–9.9, and ≥10%, respectively. Multivariable adjusted restricted cubic spline analyses showed a linear association of HbA1c with the recurrence of AF, with HbA1c 6–7.9% as the reference value (*P* < 0.001; Figure 2).

**Table 1:** Hazard ratios of atrial fibrillation recurrence after ablation by 5 levels of HbA1c as continuous variables.

**HbA1c (%)**	**Person-years of follow-up**	**No. of events**	**Rate per 100 person-years**	**Unadjusted HR (95% CI)**	**Adjusted HR (95% CI)**	***P* for trend**
<4	68	2	2.94	0.65 (0.49–0.83)	0.58 (0.51–0.72)	0.01
4–5.9	215	8	3.72	0.88 (0.71–0.98)	0.85 (0.63–0.97)	
6–7.9	705	59	8.37	1.00	1.00	
8–9.9	350	30	8.58	1.36 (1.07–1.52)	1.25 (1.03–1.48)	
≥10	247	22	8.91	1.72 (1.34–1.85)	1.51 (1.23–1.76)	

*HR, Hazard ratio; CI, Confidence interval*.

### Baseline Characteristics and Perioperative Variables Between Patients With HbA1c<7.5% and Those With HbA1c ≥ 7.5%

To investigate the impact of glycemic control on patients with VAF, given the above results, we used an HbA1c level of 7.5% as the cutoff value and then divided all patients into two groups (Group H < 7.5% and Group H ≥ 7.5%). As demonstrated, patients with HbA1c ≥ 7.5% had a higher rate of persistent AF (82.3 vs. 44.1%; *P* = 0.01) and a lower rate of metformin use (40.8 vs. 59.3%; *P* = 0.01). In addition, more patients in Group H ≥ 7.5% exhibited a higher proportion of increase in HbA1c (47.2 vs. 10.7%; *P* = 0.01), and fewer showed a lower proportion of decrease in HbA1c (52.8 vs. 89.3%; *P* = 0.01) (Table 2). Renal function, congestive heart failure and echocardiographic data were not significantly different between the two groups, nor was valvular heart disease.

**Table 2:** Preoperative clinical data comparison.

**Characters**	**Group H < 7.5% (*n* = 177)**	**Group H ≥7.5% (*n* = 142)**	***P*-value**
**Baseline data**
Age (years ± SD)	57.6 ± 7.1	58.2 ± 8.3	0.68
Male	50.2%(89)	50.0%(71)	0.96
BMI(Kg/m^2^)	24.1 ± 3.5	24.2 ± 3.3	0.82
Hypertension	24.8%(44)	29.5%(42)	0.34
Recent smoking	16.3%(29)	19.0%(27)	0.53
COPD	20.3%(36)	25.3%(36)	0.28
Cerebrovascular disease	13.5%(24)	15.4%(22)	0.62
History of CHF	22.0%(39)	31.6%(45)	0.06
NYHA III-IV	21.4%(38)	29.6%(42)	0.09
Creatinine (umol/L)	1.4 ± 0.4	1.4 ± 0.5	0.51
BUN (mg/dl)	23.1 ± 8.7	24.2 ± 9.6	0.27
BNP(pg/ml)	297.5 ± 244.3	294.8 ± 254.8	0.92
CRP(mg/L)	88.6 ± 82.2	86.9 ± 86.3	0.85
Persistent AF	44.1%(78)	82.3%(117)	0.01
**Glycemic control data**
Decrease in HbA1c	89.3%(158)	52.8%(75)	0.01
Increase in HbA1c	10.7%(19)	47.2%(67)	0.01
Insulin	32.2%(57)	38.0%(54)	0.27
Metformin	59.3%(105)	40.8%(58)	0.01
Sulfonylurea	16.9%(30)	24.6%(35)	0.09
Thiazolidinedione	2.8%(5)	5.6%(8)	0.21
DPP-4 inhibitor	11.2%(20)	16.9%(24)	0.15
GLP-1 receptor agonist	3.3%(6)	6.3%(9)	0.21
SGLT-2 inhibitor	3.3%(6)	3.5%(5)	0.95
**Echocardiographic data and 3D-CT**
LVEF (%)	46.5 ± 15.1	52.6 ± 15.8	0.78
LVEDD (mm)	50.9 ± 8.6	51.1 ± 8.7	0.81
LA diameter (mm)	48.8 ± 15.4	48.9 ± 15.6	0.95
LA volume/BSA (mL/m^2^)	117 ± 87	128 ± 96	0.36
**Valvular heart disease**
Mitral stenosis	41.8%(74)	40.1%(57)	0.76
Mitral regurgitation	30.5%(54)	28.8%(41)	0.75
Tricuspid regurgitation	46.8%(83)	42.2%(60)	0.41
Aortic stenosis	17.5%(31)	11.2%(16)	0.12
Aortic regurgitation	12.9%(23)	12.6%(18)	0.93
Combined valvular disease	35.5%(63)	33.0%(47)	0.64

*H, HbA1c (%); SD, Standard Deviations; BMI, Body mass index; COPD, Chronic obstructive pulmonary disease; CHF, congestive heart failure; NYHA, New York Heart Function Assessment; BUN, blood urea nitrogen; BNP, Brain natriuretic peptides; CRP, C-reactive protein; AF, Atrial Fibrillation; HbA1c, glycated hemoglobin; DPP-4, dipeptidyl peptidase-4; GLP-1, glucagon-like peptide-1; SGLT-2, sodium-glucose cotransporter-2; LVEF, left ventricular ejection fraction; LVEDD, left ventricular end diastolic diameter; LA, Left Atrial; BSA, body surface area*.

The perioperative clinical data of the patients are listed in Table 3. Compared with patients in Group H < 7.5%, those in Group H ≥ 7.5% had a similar hospitalization time before surgery and duration of operation. In addition, there were no statistically significant differences in ICU stay, mechanical ventilation time, cardiopulmonary bypass time, aortic cross-clamp time, radiofrequency ablation device type or concomitant valvular procedures between the two groups.

**Table 3:** Perioperative variables comparison.

**Characters**	**Group H < 7.5% (*n* = 177)**	**Group H ≥7.5% (*n* = 142)**	***P*-value**
Hospitalization time before surgery (d)	5.2 ± 2.9	7.1 ± 2.4	0.23
Hospitalization time after surgery (d)	8.1 ± 2.7	8.5 ± 3.2	0.34
ICU staying (h)	22.8 ± 4.7	25.3 ± 6.2	0.54
Mechanical ventilation time (h)	21.9 ± 3.2	22.8 ± 2.7	0.28
Emergency operation	2.2% (4)	6.3% (9)	0.06
Duration of operation (h)	3.3 ± 0.3	4.1 ± 0.7	0.52
Cardiopulmonary bypass time (min)	131.3 ± 41.0	130.1 ± 37.1	0.77
Aortic cross-clamp time (min)	95.5 ± 35.3	95.8 ± 33.3	0.93
Medtronic RA device	33.8%(60)	33.0%(47)	0.88
AtriCure RA device	66.1%(117)	66.9%(95)	0.88
**Concomitant procedures**
Mitral valve repair	19.7%(35)	21.1%(30)	0.76
Mitral valve replacement	52.5%(93)	47.8%(68)	0.41
Tricuspid valve repair	44.6%(79)	40.8%(58)	0.49
Aortic valve replacement	31.0%(55)	23.2%(33)	0.12

*H, HbA1c (%); NR, No recurrence; R, Recurrence; ICU, Intensive care unit; RA, Radiofrequency Ablation*.

### Effect of Glycemic Control on Clinical Outcomes

**Figure 3:**
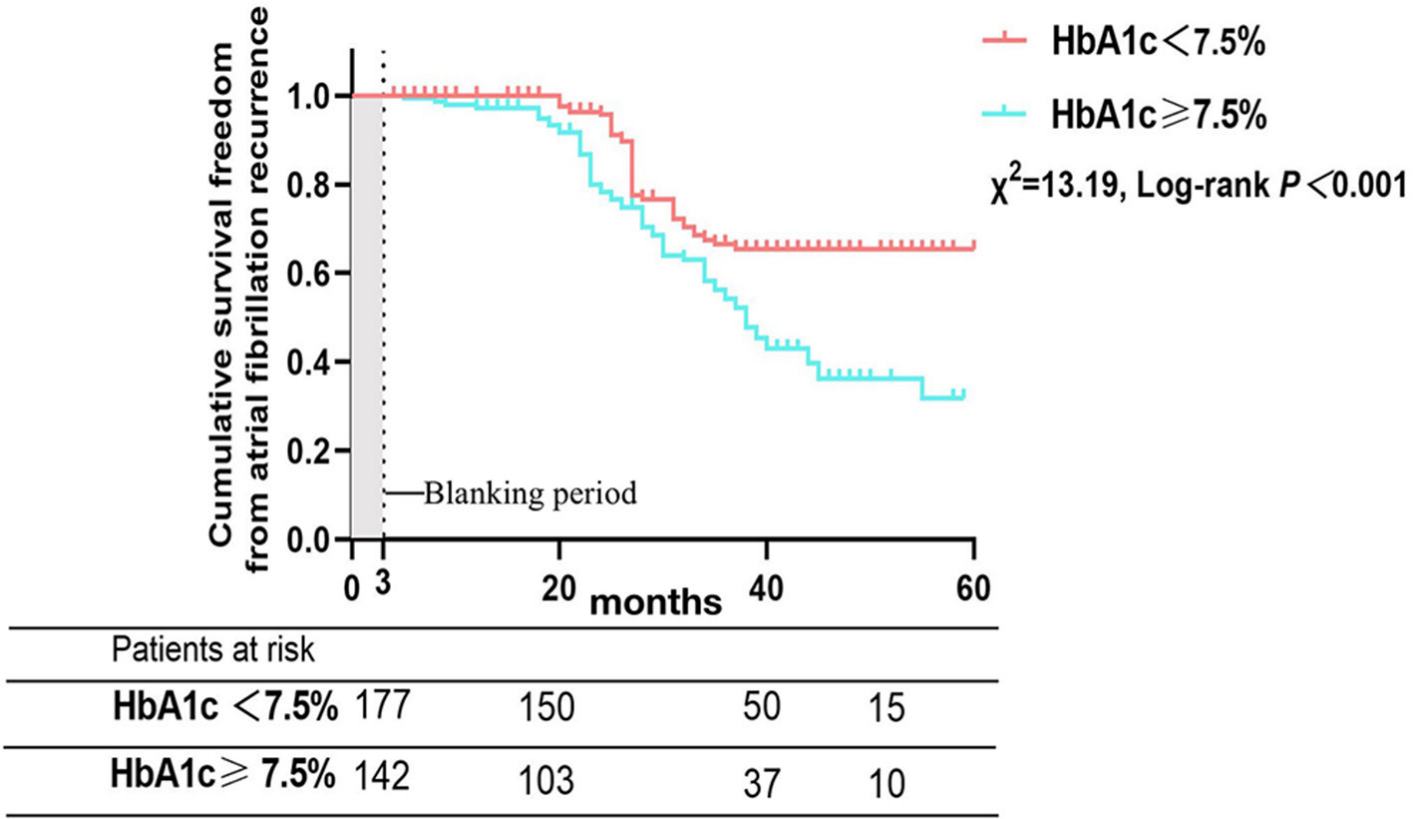
Atrial fibrillation recurrence rates after Cox-Maze IV ablation according to glycated hemoglobin at the time of operation.

**Figure 4:**
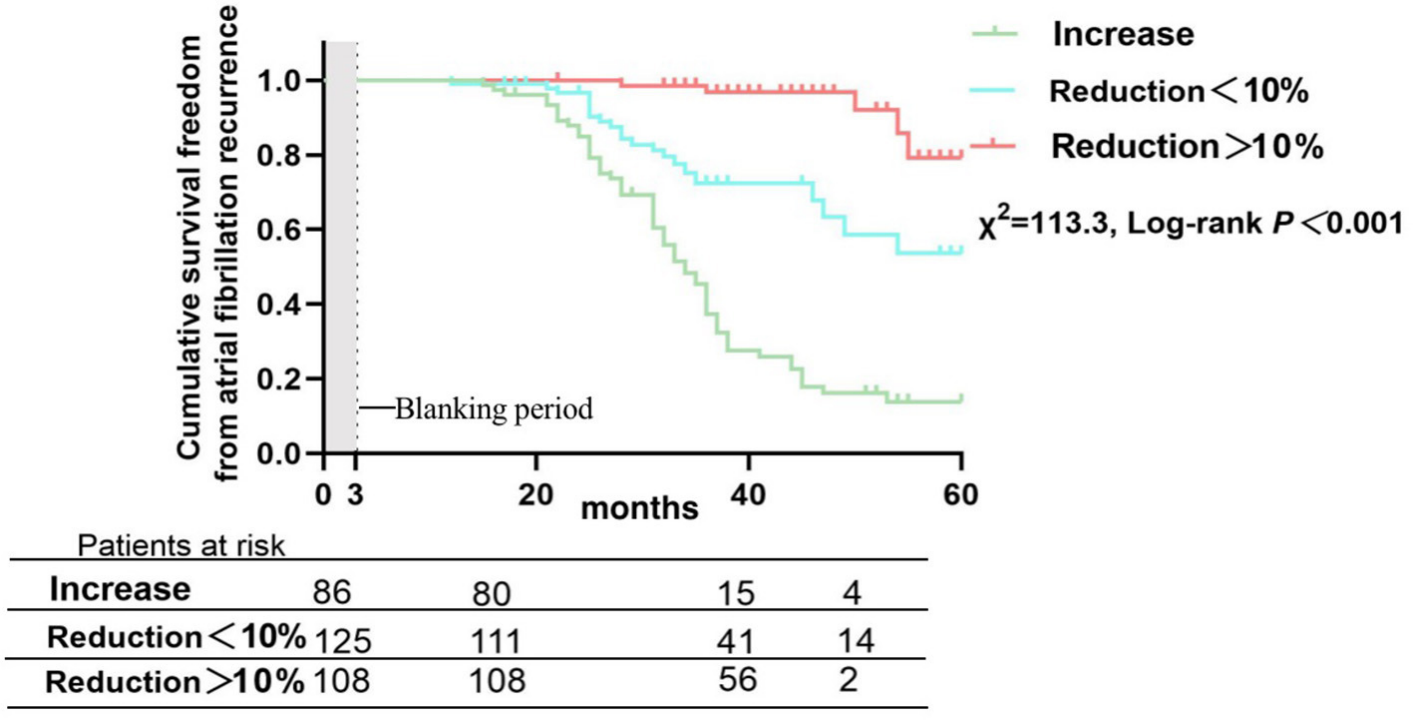
Atrial fibrillation recurrence rates after Cox-Maze IV ablation in patients divided by percentage change in glycated hemoglobin during the 12 months preoperatively.

There were 121 patients (37.9%) who developed AF recurrence, with a mean follow-up period of 36.7 ± 23.3 months. The cumulative atrial fibrillation recurrence-free survival of patients with HbA1c < 7.5% at 12, 24, 36 and 48 months was 100, 95.7, 66.5, and 65.3%, respectively, and 97.1, 78.3, 54.2, and 36.3% for patients with HbA1c ≥ 7.5%, respectively (log-rank χ^2^=13.19; *P* < 0.001) (Figure 3). In addition, patients were subsequently divided into three groups depending on the trend of HbA1c, with a threshold of a 10% reduction in HbA1c defined to reflect strict glycemic control (12), during the 12 months before the Cox-Maze IV procedure to analyze the effect of glycemic control on clinical outcomes and recurrence of AF (Group IH: increase in HbA1c, Group DH: decrease in HbA1c < 10%, Group DH10: decrease in HbA1c ≥ 10%). The cumulative atrial fibrillation recurrence-free survival was 100, 84.9, 37.2, and 16.2% for patients with an increase in HbA1c at 12, 24, 36, and 48 months, respectively; 99.0, 96.6, 72.4, and 58.5% for patients with a decrease in HbA1c <10%, respectively; and 100, 98.5, 96.8, and 91.9% for patients with a decrease in HbA1c > 10%, respectively (log-rank χ^2^=113.3; *P* < 0.001) (Figure 4).

Surgical mortality and major postoperative morbidity between the groups are shown in Tables 4, 5. For patients in Group H < 7.5% and Group H ≥ 7.5%, there was no significant difference in surgical mortality (1.6 vs. 1.4%, *P* = 0.83). Infection, renal insufficiency, re-exploration for bleeding, perioperative myocardial infarction, perioperative stroke, prolonged ventilation, continuous renal replacement therapy (CRRT), intra-aortic balloon pump (IABP) and extracorporeal membrane oxygenation (ECMO) were similar between the two groups. However, there were 15 deaths during follow-up, and the rates of all-cause mortality, cardiac mortality and rehospitalization were significantly lower in Group H < 7.5% (1.6 vs. 6.3%, *P* = 0.03; 1.1 vs. 5.6%, *P* = 0.02 and 6.2 vs. 20.4%, *P* = 0.01). The rates of stroke and hemorrhage were not significantly different between the two groups.

**Table 4:** Clinical outcomes for study cohorts.

**Characters**	**Group H < 7.5% (*n* = 177)**	**Group H ≥7.5% (*n* = 142)**	***P*-value**
**In-hospital**
Surgical mortality	1.6%(3)	1.4%(2)	0.83
Infarction	1.1%(2)	2.1%(3)	0.48
Stroke	4.5%(8)	4.9%(7)	0.86
Infection	0.5%(1)	0.7%(1)	0.88
Prolonged ventilation	2.2%(4)	1.4%(2)	0.57
Re-exploration for bleeding	6.2%(11)	6.3%(9)	0.96
CRRT	2.8%(5)	4.2%(6)	0.49
IABP	3.3%(6)	4.9%(7)	0.48
ECMO	1.6%(3)	1.4%(2)	0.83
**Follow-up**
All-cause mortality	1.6%(3)	6.3%(9)	0.03
Cardiac-cause mortality	1.1%(2)	5.6%(8)	0.02
Re-hospitalization	6.2%(11)	20.4%(29)	0.01
Stroke	6.2%(11)	4.9%(7)	0.62
Hemorrhage	2.8%(5)	4.9%(7)	0.32

*H, HbA1c (%); CRRT, continuous renal replacement therapy; IABP, intra-aortic ballon pump; ECMO, extracorporeal membrane oxygenation; MI, myocardial infarction*.

**Table 5:** Clinical outcomes for study cohorts.

**Characters**	**Number (%) of events**			***P*-value**
	**Group DH (*n* = 125)**	**Group DH10 (*n* = 108)**	**Group IH (*n* = 86)**	
**In-hospital**
Surgical mortality	1.6%(2)	0.9%(1)	2.3%(2)	0.26
Infarction	0.8%(1)	0.9%(1)	3.4%(3)	0.23
Stroke	3.2%(4)	3.7%(4)	8.4%(7)	0.74
Infection	0(0)	0(0)	2.3%(2)	0.72
Prolonged ventilation	0.8%(1)	0.9%(1)	4.6%(4)	0.16
Re-exploration for bleeding	5.6%(7)	4.6%(5)	9.3%(8)	0.24
CRRT	2.4%(3)	1.9%(2)	7.4%(6)	0.18
IABP	4.0%(5)	3.7%(4)	5.1%(4)	0.51
ECMO	2.4%(3)	0(0)	2.1%(2)	0.13
**Follow-up**
All-cause mortality	1.6%(2)	0.9%(1)	10.4%(9)	<0.01^*^
Cardiac-cause mortality	0.8%(1)	0.9%(1)	9.3%(8)	<0.01^*^
Re-hospitalization	7.2%(9)	7.4%(8)	26.7%(23)	<0.01^*^
Stroke	4.0%(5)	5.5%(6)	8.1%(7)	0.12
Hemorrhage	2.4%(3)	3.7%(4)	5.8%(5)	0.23

*Group DH, decrease in HbA1c <10%; Group DH10, decrease in HbA1c ≥ 10%; IH, increase in HbA1c; CRRT, continuous renal replacement therapy; IABP, intra-aortic ballon pump; ECMO, extracorporeal membrane oxygenation; MI, myocardial infarction*.

* *P-value reflects the comparisons of categorical variables among the three subgroups using univariate statistics (Chi-square test or Fischer's exact test where appropriate) with post hoc Bonferroni corrections*.

For patients divided into Group IH, Group DH and Group DH10, there was also no significant difference in surgical mortality (1.6 vs. 0.9 vs. 2.3%, *P* = 0.26). Infection, renal insufficiency, re-exploration, perioperative myocardial infarction, perioperative stroke, prolonged ventilation, CRRT, IABP, ECMO, stroke and hemorrhage during follow-up were similar among the three groups. However, the rates of all-cause mortality, cardiac mortality and rehospitalization were significantly different among the three groups (1.6 vs. 0.9 vs. 10.4%, *P* < 0.01; 0.8 vs. 0.9 vs. 9.3%, *P* < 0.01, and 7.2 vs. 7.4 vs. 26.7%, *P* < 0.01).

### Predictors of the Recurrence of AF

The baseline characteristics of patients who had recurrent AF are shown in Supplementary Table 1. According to univariate Cox proportional hazards analyses, a downward trend (% decrease) in HbA1c during the 12-month period before ablation was associated with markedly lower rates of AF recurrence (HR: 0.634; 95% CI: 0.463–0.834; *P* = 0.01), while an upward trend (% increase) in HbA1c was linked to higher recurrence rates (HR: 1.156; 95% CI: 1.086–1.568; *P* = 0.01). Older age was also associated with higher AF recurrence rates (HR: 1.124; 95% CI: 1.052–1.165; *P* = 0.01). HbA1c ≥ 7.5% at the time of ablation (HR: 1.254; 95% CI: 1.014–1.954; *P* = 0.01) and mitral stenosis (HR: 1.146; 95% CI: 1.037–1.583; *P* = 0.01) were also associated with higher AF recurrence rates, and the use of metformin was associated with lower rates of recurrence (HR: 0.582; 95% CI: 0.427–0.968; *P* = 0.02) (Table 6). Furthermore, according to multivariate Cox proportional hazards analyses adjusting for age, the trend of HbA1c, mitral stenosis, the trend of HbA1c before ablation and the use of metformin, the effects of age (HR: 1.015; 95% CI: 0.938–1.086; *P* = 0.08), the use of metformin (HR: 0.762; 95% CI: 0.597–1.363; *P* = 0.75) and persistent AF (HR: 1.134; 95% CI: 0.941–1.357; *P* = 0.28) were significantly eliminated (Table 6). A downward trend in HbA1c during the 12-month period before ablation (HR: 0.871; 95% CI: 0.621–0.937; *P* = 0.01) remained a significant predictor of atrial fibrillation-free status, but an upward trend in HbA1c during the 12-month period before ablation (HR: 1.123; 95% CI: 1.006–1.497; *P* = 0.01), HbA1c ≥ 7.5% (HR: 1.127; 95% CI: 1.008–1.473; *P* = 0.37) and mitral stenosis (HR: 1.089; 95% CI: 1.011–1.393; *P* = 0.01) remained significant predictors of increased AF recurrence.

**Table 6:** Hazard ratios for AF recurrence after ablation.

**Variables**	**Univariate**		**Multivariate**	
	**HR (95% CI)**	***P*-Value**	**HR (95% CI)**	***P*-Value**
Age	1.124 (1.052–1.165)	0.01	1.015 (0.938–1.086)	0.08
Gender	1.326 (0.957–1.973)	0.14		
BMI	1.012 (0.964–1.043)	0.48		
Recent smoking	1.128 (0.991–1.147)	0.62		
CHF	1.138 (0.916–1.439)	0.26		
NYHA III-IV	1.021 (0.983–1.124)	0.62		
Creatinine (umol/L)	0.968 (0.832–1.154)	0.74		
BUN (mg/dl)	1.036 (0.973–1.125)	0.11		
Persistent AF	1.368 (1.005–1.967)	0.01	1.134 (0.941–1.357)	0.28
HbA1c (%) ≥ 7.5	1.254 (1.014–1.954)	0.01	1.127(1.008–1.473)	0.01
Insulin use	1.241 (0.818–1.763)	0.26		
Metformin	0.582 (0.427–0.968)	0.02	0.762 (0.597–1.363)	0.75
Sulfonylurea	1.236 (0.967–1.828)	0.13		
Thiazolidinedione	0.829 (0.325–2.034)	0.87		
DPP-4 inhibitor	0.875 (0.643–1.046)	0.26		
GLP-1 receptor agonist	0.934 (0.435–1.956)	0.71		
SGLT-2 inhibitor	0.925 (0.356–2.754)	0.81		
Decrease in HbA1c	0.634 (0.463–0.834)	0.01	0.871 (0.621–0.937)	0.01
Increase in HbA1c	1.156 (1.086–1.568)	0.01	1.123 (1.006–1.497)	0.01
LVEF	0.992 (0.967–1.024)	0.89		
LA diameter	0.936 (0.835–1.135)	0.37		
LA volume	0.925 (0.798–1.096)	0.26		
Medtronic RA device	0.946 (0.857–1.105)	0.67		
AtriCure RA device	0.994 (0.872–1.125)	0.36		
Mitral stenosis	1.146 (1.037–1.583)	0.01	1.089 (1.011–1.393)	0.01
Mitral regurgitation	0.925 (0.731–1.367)	0.78		
Tricuspid regurgitation	0.836 (0.672–1.132)	0.69		
Aortic stenosis	0.979 (0.831–1.148)	0.22		
Aortic regurgitation	0.984 (0.741–1.098)	0.37		
Combined valvular disease	0.991 (0.874–1.148)	0.59		

*HR, Hazard ratio; CI, Confidence interval; BMI, Body mass index; CHF, congestive heart failure; NYHA, New York Heart Function Assessment; BUN, blood urea nitrogen; AF, Atrial Fibrillation; HbA1c, glycated hemoglobin; DPP-4, dipeptidyl peptidase-4; GLP-1, glucagon-like peptide-1; SGLT-2, sodium-glucose cotransporter-2; LVEF, left ventricular ejection fraction; LA, Left Atrial; RA, Radiofrequency Ablation. Univariate and multivariate Cox proportional hazards models were developed using logistic regression analysis adjusted for confounders (age, body mass index, hypertension, recent smoking, history of congestive heart failure, blood urea nitrogen, C-reactive protein, left ventricular ejection fraction)*.

## Discussion

A key finding of this study was that improved preoperative glycemic control in valvular atrial fibrillation patients with DM is related to a marked reduction in AF recurrence rates after heart valve surgery concomitant with Cox-Maze IV ablation. For patients with HbA1c < 7.5%, the cumulative atrial fibrillation recurrence-free survival at 12, 24, 36, and 48 months were higher compared with patients who had HbA1c ≥ 7.5% at ablation (log-rank χ^2^=13.19; *P* < 0.001). Otherwise, the preoperative trend in HbA1c is a dramatic predictor of AF recurrence. DM is associated with increases in both cardiovascular events and cardiovascular mortality (13), and a significant number of events are due to embolization associated with AF (14, 15). DM can also lead to a high risk of AF, and a meta-analysis that included 11 studies and 1,686,097 patients reported that DM can increase the risk of AF to more than 34% (1). The Framingham Heart Study also showed that DM can increase the risk of AF, with odds ratios of 1.6 for women and 1.4 for men (16). The pathogenesis between DM and AF is complicated and includes autonomic nervous system disorder, hyperinsulinemia, chronic inflammation (17), and structural and electromechanical remodeling (18, 19). An increasing number of studies have shown that glycemic control in diabetic patients is a crucial therapeutic strategy for decreasing the AF recurrence rate among patients with catheter ablation (8, 20). Mohanty et al. (21) reported that patients who had metabolic syndrome and underwent catheter ablation had higher recurrence rates of AF (39 vs. 32%; *p* = 0.005). Hence, in an era of elevated consciousness of the beneficial effects of lifestyle adjustments and the optimal therapy strategy before ablation, our study contributed to this field by demonstrating that more aggressive monitoring and control of HbA1c before ablation in DM patients with AF followed by heart valve surgery concomitant with the Cox-Maze IV procedure can reduce the recurrence of AF.

In the present study, there was no significant difference in surgical mortality, infection, renal insufficiency, re-exploration for bleeding, perioperative myocardial infarction, perioperative stroke, prolonged ventilation, CRRT, IABP or ECMO among the groups (Group H < 7.5%/Group H ≥ 7.5% and Group IH/Group DH/Group DH10). However, there were 15 deaths during follow-up, and the rates of all-cause mortality, cardiac mortality and rehospitalization were significantly lower in Group H < 7.5% (1.6 vs. 6.3%, *P* = 0.03; 1.1 vs. 5.6%, *P* = 0.02 and 6.2 vs. 20.4%, *P* = 0.01). In addition, the rates of the above three clinical outcomes were also significantly lower in Group DH and Group DH10 than in Group IH (1.6 vs. 0.9 vs. 10.4%, *P* < 0.01; 0.8 vs. 0.9 vs. 9.3%, *P* < 0.01, and 7.2 vs. 7.4 vs. 26.7%, *P* < 0.01). These results indicate that patients with poor preoperative glycemic control are more likely to experience rehospitalization and postoperative cardiac death, which may result from the higher recurrence of AF. Therefore, it is urgently necessary to improve the HbA1c level of patients prior to ablation to reduce mortality.

In multivariate analysis, our study demonstrated that an increase in HbA1c during the 12 months prior to ablation (HR: 1.123; 95% CI: 1.006–1.497; *P* = 0.01), HbA1c ≥ 7.5% at the time of operation (HR: 1.127; 95% CI: 1.008–1.473; *P* = 0.37) and mitral stenosis (HR: 1.089; 95% CI: 1.011–1.393; *P* = 0.01) were significant predictors of recurrent AF. HbA1c ≥ 7.5% at the time of operation and an upward trend of HbA1c before ablation reflected poor glycemic control before surgery, which can increase autonomic dysfunction, as well as inflammatory reactions (17) and structural remodeling (18), leading to the recurrence of AF. There were few studies examined the effect of HbA1c control at the time of ablation on AF recurrence, and the sample size were very small. In the Atherosclerosis Risk in Communities study (14), the researchers showed that, regardless of whether patients had DM, HbA1c was independently associated with incident AF; furthermore, Donnellan et al. (8) reported that the 12-month trend in HbA1c was related to the recurrence of AF in patients who underwent catheter ablation and that patients with an increase in the HbA1c level during the 12 months before ablation had a higher recurrence rate of AF (*P* < 0.001). The ARREST-AF study reported that control of preablation risk factors, such as blood pressure and glycemic modification, can decrease the recurrence of AF (9.7 vs. 32.9%; *P* < 0.001) (22), but only 26 patients with DM were included in the study. Moreover, it has previously been shown that mitral stenosis is a predictor of AF recurrence (11), which was consistent with the current study. Our study has added prominent evidence to the field of AF recurrence risk factor modification, which could improve the rates of successful AF ablation.

There are several limitations to our study. First, the study has the usual limitations of retrospective investigations, although all consecutive patients who met the inclusion criteria were enrolled. Furthermore, we did not study other atrial tachyarrhythmias in patients who were AF free. In addition, owing to the lack of some clinical data, we could not report the trend of HbA1c during follow-up, which needs further study. Otherwise, we collected at least two HbA1c measurements at our institution. Limited by the study design, the time span for HbA1c before surgery may be longer in some patients, so we had calculated the HbA1c trend depending on the initial and perioperative values, which needs the futher investigate.

## Conclusions

Improved glycemic control prior to Cox-Maze IV ablation in DM patients with valvular atrial fibrillation is associated with a dramatic reduction in atrial fibrillation recurrence rates after ablation. Our study suggests that maintaining a downward trend in HbA1c during the 12-month period before ablation and an HbA1c value < 7.5% at the time of ablation is more important in reducing the recurrence of AF. The glycemic control plan for patients planning to undergo Cox-Maze IV ablation should start early, and a diabetes specialist can play a crucial role in this process.

## Data Availability Statement

The raw data supporting the conclusions of this article will be made available by the authors, without undue reservation.

## Author Contributions

ZP and KH wrote the main manuscript text. RZ and YL prepared figures. YY and XY prepared tables. All authors reviewed the manuscript. All authors contributed to the article and approved the submitted version.

## Conflict of Interest

The authors declare that the research was conducted in the absence of any commercial or financial relationships that could be construed as a potential conflict of interest.

## Publisher's Note

All claims expressed in this article are solely those of the authors and do not necessarily represent those of their affiliated organizations, or those of the publisher, the editors and the reviewers. Any product that may be evaluated in this article, or claim that may be made by its manufacturer, is not guaranteed or endorsed by the publisher.
