# Supplementary material for: Effect of Preablation Glycemic Control on Outcomes of Atrial Fibrillation Patients With Diabetes Mellitus Following Valvular Surgery Combined With the Cox-Maze IV Procedure
Source: Front Cardiovasc Med. 2022 May 11;9:898642. doi: 10.3389/fcvm.2022.898642 (PMC9130630; doi:10.3389/fcvm.2022.898642)
Supplement: Supplementary file 1 [file Table_1.DOCX]

Supplement Table 1. Preoperative clinical data comparison

| Characters | Group NR(n=198) | Group R(n=121) | *P* value |
| --- | --- | --- | --- |
| **Baseline data** | | | |
| Age(years±SD) | 58.2±6.2 | 69.6±7.9 | 0.01 |
| Male | 46.3%(92) | 56.2%(68) | 0.09 |
| BMI(Kg/m^2^) | 23.4±3.9 | 24.1±4.6 | 0.41 |
| Hypertension | 32.3%(64) | 28.2%(34) | 0.42 |
| Recent smoking | 19.2%(38) | 14.9%(18) | 0.33 |
| COPD | 23.7%(47) | 20.7%(25) | 0.52 |
| Cerebrovascular disease | 12.6%(25) | 17.4%(21) | 0.24 |
| History of CHF | 24.2%(48) | 29.7%(36) | 0.27 |
| NYHA III-IV | 22.2%(44) | 29.7%(36) | 0.17 |
| [Creatinine](C:/Users/surface/AppData/Local/youdao/dict/Application/8.9.3.0/resultui/html/index.html#/javascript:;) (umol/L) | 1.3±0.2 | 1.6±0.7 | 0.55 |
| BUN (mg/dl) | 22.3±9.7 | 23.6±10.1 | 0.13 |
| BNP(pg/ml) | 263.4±229.7 | 349.9±270.2 | 0.11 |
| CRP(mg/L) | 70.9±72.1 | 86.4±64.7 | 0.24 |
| CHA2DS2-VASC | 3.4±1.3 | 4.1±1.8 | 0.36 |
| Persistent AF | 56.4%(112) | 68.6%(83) | 0.03 |
| **Glycemic control data** | | | |
| HbA1c (%) | 6.4±1.6 | 7.9±1.7 | 0.01 |
| decrease in HbA1c | 91.9%(182) | 42.1%(51) | 0.01 |
| increase in HbA1c | 5.6%(11) | 61.9%(75) | 0.01 |
| Insulin | 31.3%(62) | 40.5%(49) | 0.09 |
| Metformin | 55.6%(110) | 43.8%(53) | 0.04 |
| Sulfonylurea | 17.2%(34) | 25.6%(31) | 0.06 |
| Thiazolidinedione | 3.5%(7) | 4.9%(6) | 0.53 |
| DPP-4 inhibitor | 12.1%(24) | 16.5%(20) | 0.26 |
| GLP-1 receptor agonist | 3.5%(7) | 6.6%(8) | 0.21 |
| SGLT-2 inhibitor | 2.5%(5) | 4.9%(6) | 0.24 |
| **Echocardiographic data and 3D-CT** | | | |
| LVEF（%） | 45.3±14.3 | 53.7±16.3 | 0.58 |
| LVEDD(mm) | 51.1±8.7 | 50.7±8.6 | 0.68 |
| LA diameter (mm) | 45.8±14.3 | 53.7±16.3 | 0.16 |
| LA volume/BSA (mL/m^2^) | 123±94 | 131±108 | 0.24 |
| **Valvular heart disease** | | | |
| Mitral stenosis | 27.7%(55) | 62.8%(76) | 0.01 |
| Tricuspid [regurgitation](C:/Users/surface/AppData/Local/youdao/dict/Application/8.10.3.0/resultui/html/index.html#/javascript:;) | 40.9%(81) | 51.2%(62) | 0.07 |
| Aortic stenosis | 14.3%(28) | 15.7%(19) | 0.71 |
| Aortic [regurgitation](C:/Users/surface/AppData/Local/youdao/dict/Application/8.10.3.0/resultui/html/index.html#/javascript:;) | 12.7%(25) | 13.1%(16) | 0.87 |
| Combined valvular disease | 33.3%(66) | 36.9%(44) | 0.58 |

NR:No recurrence;R:Recurrence;SD:Standard Deviations;BMI:Body mass index; COPD:Chronic obstructive pulmonary disease; CHF:congestive heart failure; NYHA:New York Heart Function Assessment; BUN:blood urea nitrogen; BNP:Brain natriuretic peptides ;CRP:C-reactive protein;AF:Atrial Fibrillation; HbA1c:glycated hemoglobin; DPP-4:dipeptidyl peptidase-4; GLP-1:glucagon-like peptide-1; SGLT-2:sodium-glucose cotransporter-2; LVEF: left ventricular ejection fraction;LVEDD:left ventricular end diastolic diameter; LA:Left Atrial;BSA, body surface area
